# How Circular Can the Chemical Industry Sector Be(come)?

**DOI:** 10.1002/gch2.202500358

**Published:** 2025-10-26

**Authors:** Leonie Barner

**Affiliations:** ^1^ School of Chemistry and Physics Faculty of Science Centre for Environment and Society Queensland University of Technology 2 George St Brisbane QLD 4000 Australia

**Keywords:** bioeconomy, chemical industry, circular economy, circularity indicators, planetary boundary system, SDG

## Abstract

The current perspective discusses the urgent need for the chemical industry to become more circular by adopting principles of circular economy. Importantly, many chemical products do not fit into the mainstream circular economy scheme that relies on a circular flow of resources, by recovering, retaining or adding to their value as many chemical products are intentionally dispersed into the environment. However, the chemical industry is a key sector that will support the return of the seven transgressed planetary boundaries to the safe operating space. Already defined circularity indicators – as published in the ISO 59000 family of standards – applicable to the chemical industry are discussed and new circularity indicators are introduced. In addition, the introduction of a waste framework with Scope 1, 2, 3 waste categories as a reporting tool is briefly discussed as an enhanced focus on waste generated along the value chain could improve the circular material use rate that currently is below 7%.

## Graphical abstract

The current perspective discusses the interconnectivity between the chemical industry, circular economy, bioeconomy, and the planetary boundaries framework. Circularity indicators specific to the chemical industry are discussed that should help the chemical sector to become more circular and therefore contribute to a sustainable future.

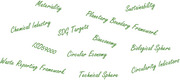

## 1 Introduction

The number of stakeholders in the chemical industry, governments as well as in academia who realize the urgency of addressing climate change, biodiversity loss, and natural resource depletion is steadily growing. One pathway ahead for an environmental, social, and economic sustainable chemical industry is the adoption of the principles of the circular economy. However, this opens the fundamental question of ‘How circular can the chemical industry sector become and how can circularity specifically for the chemical industry be measured?’. Answering these questions will support the chemical industry to transform into a truly circular sector.

## 2 The Planetary Boundary Framework

In 2009, Earth system and environmental scientists introduced an evidence‐based model to describe and define a safe operating space for the Earth System.^[^ ^1^ ^]^ They defined nine interlinked planetary boundaries: Climate change, ocean acidification, stratospheric ozone depletion, biogeochemical flow (nitrogen and phosphorus), global freshwater use, change in land use, biodiversity loss, atmospheric aerosol loading, and chemical pollution. Since its introduction, the planetary boundaries framework has been updated and extended including parameters to assess when the safe operating space of the boundaries are transgressed. In 2015, the planetary boundary of ‘chemical pollution’ was changed to ‘novel entities’, but no boundary for ‘novel entities’ was defined.^[^ ^2^ ^]^ However, in 2022, Persson et al. published an analysis showing that the safe operating space for novel entities clearly has been transgressed.^[^ ^3^ ^]^ In recent updates in 2023 and 2024, Richardson et al. found that six of the nine boundaries are transgressed and that the Earth is therefore well outside of the safe operating space for humanity.^[^ ^4^, ^5^ ^]^ They also updated the definition for ‘novel entities’ that “is now restricted to include only entities that, in the absence of the anthroposphere, are not present in the Earth system”. These entities include synthetic chemicals and substances, anthropogenically mobilized radioactive materials (such as nuclear waste and nuclear weapons), and human modification of evolution. Critically, Richardson et al. define the planetary boundary for novel entities as 0 (zero!) release of untested synthetics into the Earth system. Currently, the boundary ‘novel entities’ is in the high‐risk zone. Importantly, we need to acknowledge that the production and use of chemicals are influencing all planetary boundaries and that we need to return to or stay in the safe operating space of all nine planetary boundaries. The abovementioned reports from 2023 and 2024 have now been superseded by the ‘Planetary Health Check 2025’ report stating that now seven of the nine boundaries have been transgressed.^[^ ^6^ ^]^ The boundaries of novel entities, climate change, biosphere integrity, land system change, freshwater change, biogeochemical flows, and ocean acidification have all been transgressed by human activities. I highly recommend the ‘Planetary Health Check 2025’ report as a scientific‐based source for information about the state of our planet, key drivers that change planetary boundaries, tipping points of the Earth system, humanity's impact on the Earth system, and interaction of planetary boundaries as well as open questions that still need to be analyzed and scrutinized by science.

## 3 Definition of Circular Economy

In 2024, the ISO 59000 family of standards for circular economy was published for the first time comprising standards that contain definitions for circular economy and circularity indicators among others. The ISO 59000 standard defines circular economy as an “economic system that uses a systemic approach to maintain a circular flow of resources, by recovering, retaining or adding to their value, while contributing to sustainable development”. The ISO standard also defines sustainable development by using the definition from the 1987 Brundtland report (Our Common Future) as a “development that meets the environmental, social, and economic needs of the present without compromising the ability of future generations to meet their own needs”. The Brundtland report also stresses that “the sustainability of ecosystems on which the global economy depends must be guaranteed”.^[^ ^7^ ^]^

ISO 59020 – Measuring and assessing circularity performance – details how to measure and assess circularity performance and sustainability impacts and applies standard indicators and complementary methods.^[^ ^8^ ^]^ Standard ISO59020 references the UN Agenda 2030, the Sustainable Development Goals (SDGs) as well as the planetary boundaries framework and highlights that the quality and resilience of the ecosystem need to be secured. The standard also states that more detailed circularity assessment standards appropriate for individual sectors need to be developed. The state of circular economy is assessed using mandatory and optional circularity indicators. In addition, core circularity indicators relating to energy, water, and economics are defined (**Table** **1**). The terms circular economy and circularity are often used in a synonymous way. However, the standard defines circularity as the “degree of alignment with the principles for a circular economy”, i.e., as a measure of circular economy and not circular economy itself. The ISO59020 standard applies a materiality approach to measuring circularity, i.e., an approach that identifies the issues of the most importance to a business or sector. In addition, the standard states that complementary methods to measure and assess sustainability impacts can be used. The ISO59020 standard also emphasizes the importance of a life cycle perspective.

**Table 1:** ISO59020 core circularity indicators.^[^ ^8^ ^]^

Indicator category	Mandatory / optional	Core circularity indicator
Resource Inflow	Mandatory	Average reuse content of an inflow
Resource Inflow	Mandatory	Average recycled content of an inflow
Resource Inflow	Mandatory	Average renewable content of an inflow
Resource Outflow	Optional	Average lifetime of product or material
Resource Outflow	Mandatory	Percent of actual reused products and components derived from outflow
Resource Outflow	Mandatory	Percent of actual recycled material derived from outflow
Resource Outflow	Mandatory	Percent of actual recirculation of outflow in the biological cycle
Energy	Optional	Average percent of energy consumed that is renewable energy
Water	Optional	Percent of water withdrawal from inflow circular sources
Water	Optional	Percent of water discharge in accordance with quality requirements
Water	Optional	Ratio (on‐site or internal) water reuse or recirculation
Economic	Optional	Material productivity
Economic	Optional	Resource intensity index
*Social*	*No social core circularity indicator defined*	

**Figure 1:**
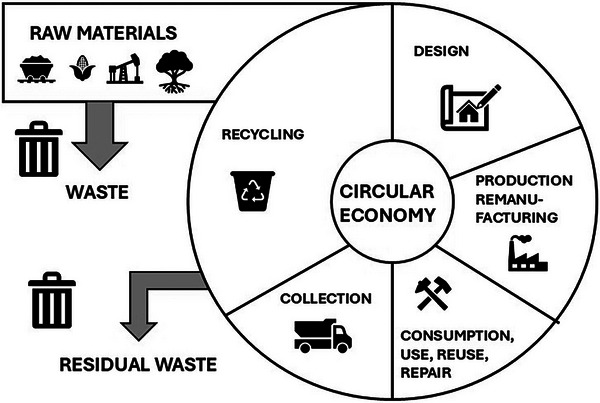
Common schematic representation of the circular economy concept including the waste stream resulting from the extraction of raw materials.

A frequently used concept of circular economy was developed by the Ellen MacArthur Foundation and describes the circular economy as “a system where materials never become waste and nature is regenerated. In a circular economy, products and materials are kept in circulation through processes like maintenance, reuse, refurbishment, remanufacture, recycling, and composting. The circular economy tackles climate change and other global challenges, like biodiversity loss, waste, and pollution, by decoupling economic activity from the consumption of finite resources.” The Ellen MacArthur Foundation visualizes the circular economy in a so‐called butterfly diagram with two separate cycles, i.e., the biological cycle and the technical cycle. In addition, they defined three design‐driven principles, i.e., eliminate waste and pollution, circulate products and materials (at their highest value), and regenerate nature.^[^ ^9^ ^]^ However, I argue that these three principles should be complemented by a fourth principle: ‘design‐out overconsumption’ to better address the ever increasing amount of resources that are extracted and the generation of waste that is caused by it. **Figure** **1** depicts one of the commonly used schematics of the circular economy with the addition of the waste stream that results from the extraction of raw materials. However, many products of the chemical industry are not well represented by this scheme as they are nondurable or are intentionally dispersed into the environment and therefore cannot be part of this circular scheme.

Other definitions of circular economy were developed as well as demonstrated by Kirchherr et al. who recently analyzed that 221 definitions of circular economy can be found in peer‐reviewed literature.^[^ ^10^ ^]^

## 4 The Circularity Gap Report

Since 2019, the Circle Economy Foundation publishes the annual Circularity Gap Report analyzing the global state of circularity.^[^ ^11^ ^]^ The report measures circularity by analyzing the material flows of economic systems and calculates the circularity metric as the proportion of secondary materials out of an economy's total material consumption, i.e., raw or primary materials consumption plus secondary material consumption. A high value of this circularity metric means that more secondary materials are used and substitute primary raw material. However, the 2025 Circularity Gap Report found that the value of the global circularity metric declined from 9.1% in 2018 to 6.9%, in conjunction with an increase in material consumption overall.

In 2022, the circular material use rate (CMUR) in the European Union was 11.5%, with the Netherlands leading at 27.5%, and Finland at just 0.6%. The EU has set the goal to double their circular material use rate to 23.2% by 2030 but is not on track to reach this target.^[^ ^12^ ^]^ In 2019, the Australian circularity rate was 5.1% and the theoretical circularity maximum was assessed at 45.6% by CSIRO.^[^ ^13^ ^]^ The question remains how high an achievable circular material use rate or global circularity material rate and overall material consumption could be while still guaranteeing a sustainable future and reversing the transgression of planetary boundaries. In addition, the state of circular economy should not be assessed by just using one indicator as one circularity indicator alone is not sufficient to represent the complexity of circular economy. Also, industry specific circularity indicators are needed to drive the establishment of circular economy.

## 5 The Chemical Industry

Chemistry is a central science that on one hand contributes to the transgression of planetary boundaries, but on the other side can also significantly contribute to solving the fundamental challenges of sustainability and support the shift back to safe planetary boundaries. In 2019, Keijer et al. introduced the concept of circular chemistry and defined twelve principles that cover aspects of chemistry, economy, policy, and environmental science highlighting the multidisciplinary nature of this concept.^[^ ^14^ ^]^ Overall, three chemical concepts exist that address sustainability, i.e., green chemistry, sustainable chemistry, and circular chemistry, but they focus on different aspects of (environmental) sustainability and the circular economy.^[^ ^15^ ^]^

There are three SDGs that specifically mention the impact of chemicals and define targets in relation to chemicals and hazardous materials.^[^ ^16^ ^]^ SDG 3 – Good health and well‐being – addresses the mortality from environmental pollution and mandates to “reduce the number of deaths and illnesses from hazardous chemicals and air, water and soil pollution and contamination” (Target 3.9). SDG 6 – Clean water and sanitation – defines Target 6.3. as “by 2030, improve water quality by reducing pollution, eliminating dumping and minimizing release of hazardous chemicals and materials, halving the proportion of untreated wastewater and substantially increasing recycling and safe use globally”. Another important chemistry‐related SDG target is included in SDG 12 – Responsible consumption and production, i.e., Target 12.4 – responsible management of chemicals and waste. Target 12.4. is defined as: “By 2020, achieve the environmentally sound management of chemicals and all wastes throughout their life cycle, in accordance with agreed international frameworks, and significantly reduce their release to air, water and soil in order to minimize their adverse impacts on human health and the environment”. It must be noted that this target was supposed to be achieved by 2020. However, we have failed to achieve this important target.^[^ ^17^ ^]^

The chemical industry engages in the manufacture and sale of chemicals and chemical products, including basic chemicals, fertilizers and nitrogen compounds, polymers and synthetic rubber, pesticides and other agrochemical products, paints, varnishes, coatings, printing inks, soaps, detergents, cleaning and polishing products, perfumes and toilet preparations, explosives and pyrotechnic products, glues, essential oils, and chemical products, as well as human‐made fibers. The chemical industry also provides important reagents for the pharmaceutical industry. Traditionally, the chemical industry uses raw materials such as fossil fuels, water, minerals, and metals to produce their products. In 2022, the global value of chemical consumption was approximately US$ 5.72 trillion and is estimated to grow even further.^[^ ^18^ ^]^

**Figure 2:**
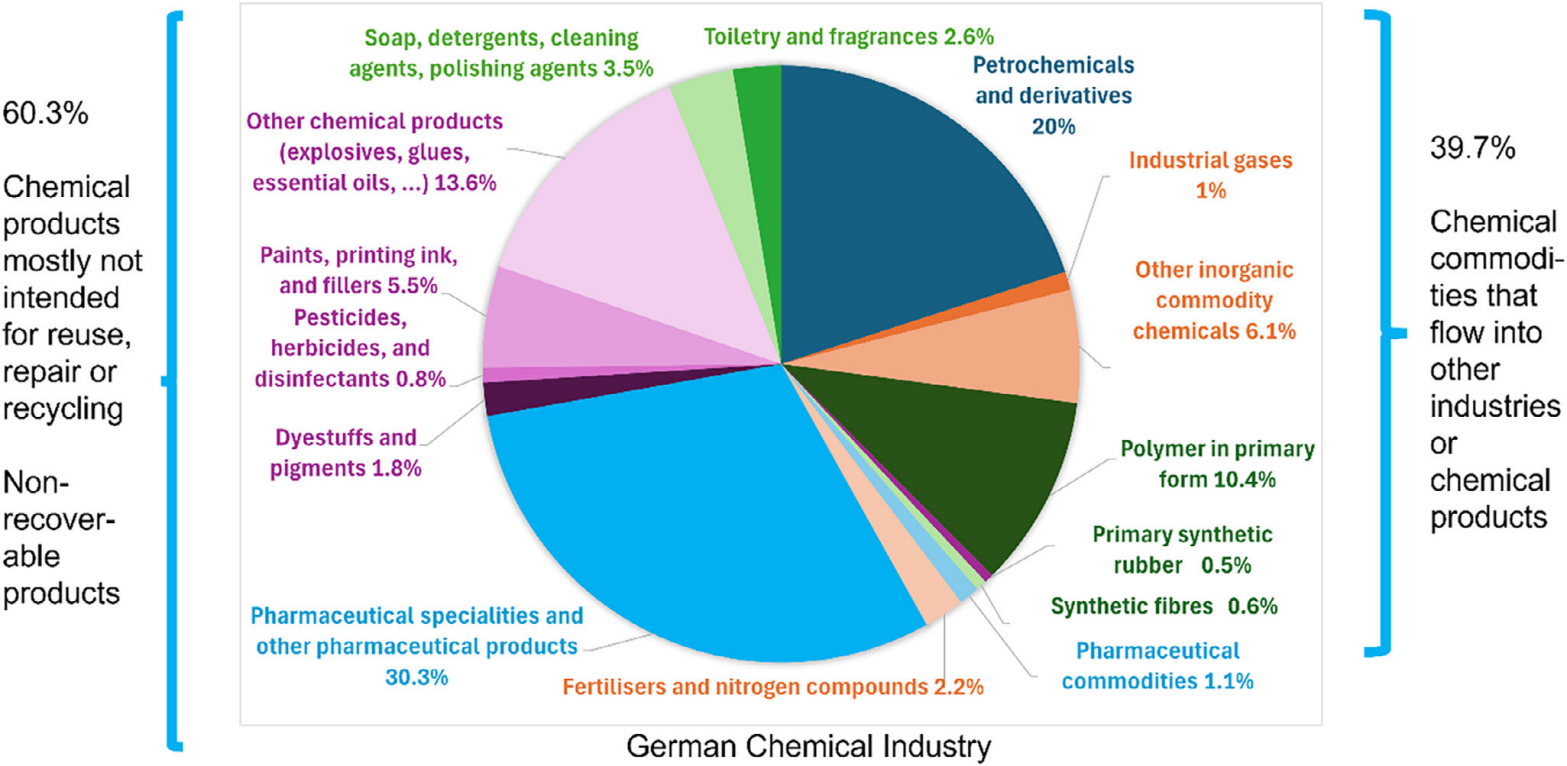
Subdivisions of the German chemical industry and market share in 2023.

Germany has a long history of a diverse chemical industry and will therefore be used as a case study in the current work. Inspection of the product portfolio of the German chemical industry^[^ ^19^ ^]^ shows that a wide range of products, i.e. materials, are challenging to be kept in circulation via maintenance, reuse, refurbishment, remanufacture, recycling, and composting as they are designed to be dispersed into the environment, i.e. products such as pesticides and fertilizers, products that are intended for human consumption such as nondurable products (e.g., soaps and detergents), or are raw materials for other industry sectors (e.g. pharmaceuticals, paper, steel, or aluminum production) (**Figure** **2**). In 2023, 60.3% of chemical products were mostly not intended for reuse, repair, or recycling and were non‐durable products. 39.7% were chemical commodities that flow into other industries or chemical products.

Therefore, the maximum achievable (material) circularity rate for the chemical sector might be significantly lower than for other industry sectors. The products of the chemical sector with the highest potential (and urgency) to become circular are polymers/plastics and synthetic rubbers as well as synthetic fibers. In 2023, these chemical products made up ≈11.5% of all chemical products produced in Germany. Therefore, setting the maximum CMUR value for the chemical industry close to 12% based on 2023 levels would be a meaningful target, however keeping in mind that the amount of polymers and plastics used globally might grow considerably over the next decades. Unfortunately, the recovery and recycling rates of plastics and fibers are notoriously low. Therefore, a concerted collaboration of all stakeholders along the polymer/plastics and synthetic fiber life cycle would be necessary to achieve the maximum CMUR value. P. Fantke and co‐workers have summarized literature about chemicals of concern in plastics and suggest five policy recommendation that would help plastics to become more circular.^[^ ^20^ ^]^ A. Paletta et al. analyzed the barriers and challenges for plastics in a circular economy using Italy as a case study. They highlight that innovative solutions are needed to achieve circular economy of plastics.^[^ ^21^ ^]^ In addition, the use of intentionally added microplastic in products such as fertilizers, plant protection products, cosmetics, household and industrial detergents, cleaning products, paints, and products used in the oil and gas industry should be banned worldwide. The European Union has restricted the use of intentionally added microplastics in October 2023 with transition periods up to 2035.^[^ ^22^ ^]^

If material circularity in the chemical industry can only be achieved for some of their products, we need to turn our attention to the circular economy design criteria ‘eliminate waste and pollution’ as well as ‘regenerate nature’ and develop carefully selected circularity indicators to assess these criteria following the materiality approach from the ISO59020 standard. One reason why the material circularity indicator is so low is the substantial increase in consumption of raw and primary materials due to an increase in the global population as well as an increase in overall wealth and access to modern products.

It is well‐known that greenhouse gas emissions must be substantially reduced to achieve the return of the planetary boundary of climate change to the safe operating zone. The chemical industry can contribute significantly by electrifying their production and the use of renewable energy (however, it should be noted that establishing renewable energy consumes a lot of raw materials). In addition, the planetary boundary for novel entities, i.e., zero release of untested novel entities (including chemicals), should be used as an indicator for the circular economy for the chemical industry (**Table** **2**). Unfortunately, only a small number of chemicals have been subjected to a safety assessment so far,^[^ ^3^ ^]^ and safety studies need to be extended to assess “cocktail effects” of chemical mixtures in the environment.^[^ ^4^ ^]^ The planetary boundary of novel entities also relates to important international conventions such as the Rotterdam Convention (importation of hazardous chemicals), the Basel Convention (movement of hazardous waste and other chemicals between nations), and the Stockholm Convention (eliminate or restrict the production and use of persistent organic pollutants). Therefore, the number of chemical companies that contribute to and countries that meet these commitments should be as high as possible and is reflected in the target of the Sustainable Development Goal 12.4 (responsible management of chemicals and waste) – which should have been achieved by 2020 but is still not accomplished. The chemical industry should also support the development and implementation of technologies to clean‐up pollution in the environment such as plastic pollution and PFAS to support the regeneration of nature. Importantly, the developed clean‐up solutions should be made available to countries of the Global South at low or no costs. The chemical industry sector has already started to identify renewable, biobased materials to substitute fossil fuel‐based chemicals and is embracing the developing bioeconomy sector. However, the impact of bioeconomy on the planetary boundaries needs to be carefully considered and scrutinized to the same extent as fossil fuel‐based materials.

**Table 2:** Additional and new circularity indicators for the chemical industry related to the planetary boundary framework.^[^ ^4^ ^]^

Earth System Process	Control variable(s)	Planetary Boundary	Preindustrial Holocene base value	Upper end of zone of increasing risk	Current value of control variable
Novel Entities	Percentage of synthetic chemicals released to the environment without adequate safety testing	0	0	NA	Transgressed
New Circularity Indicator – Safe Chemicals (suggested by this work)					
Percent of safe chemicals released over all chemicals released, indicator should be as high as possible, achieving 100% as soon as possible, also reflects SDG Targets 3.9, 6.3, 12.4					
Climate Change	Atmospheric CO_2_ concentration (ppm CO_2_)	350	280	450	420
New Circularity Indicator – CO_2_ sequestered (permanently) (suggested by this work)					
The amount of CO_2_ sequestered by chemical products along their life cycle (cradle‐to‐grave, net benefit), ideally CO_2_ should be sequestered into products with a long lifespan					
Biogeochemical flows P	P (regional): P flow from fertilizers to erodible soils	6.2 Tg of P year^−1^ mined and applied to erodible (agricultural) soils. Boundary is a global average, but regional distribution is critical for impacts.	0 Tg of P year^−1^	*regional*: 11.2 Tg of P year^−1^	*regional*: 17.5 Tg of P year^−1^
Additional ISO circularity indicator – Nutrient extracted from discharged water					
Percent of materials recovered from wastewater at End‐of‐Life and redirected to production					

## 6 New and Existing Circularity Indicators for the Chemical Sector

The chemical industry needs circularity indicators that are meaningful to its products and support and drive the circular and sustainable development of the sector. As explained above, the circular economy standard applies a materiality approach to measuring circularity, i.e., an approach that identifies the issues of the most importance to a business or sector. All mandatory core circularity indicators from ISO59020 as listed in Table 1 are of relevance to the chemical industry.

The circularity indicator that measures the percentage of actual recirculation of outflow in the biological cycle is very relevant for products of the chemical industry that are released into the environment intentionally. Importantly, the standard defines this circularity indicator as “the fraction of outflow that is recirculated at end of life for safe return to the biosphere (biodegradation) and meets the qualifying conditions for recirculation (e.g., composting or anaerobic digestion)”. There are several chemical products that are marketed as biodegradable and therefore would be assessed using the aforementioned indicator. However, the recirculation indicator must be applied with caution as chemical products can be biodegradable and fossil‐based, i.e. there is a crossover between the technical and biological sphere. For example, polyvinyl alcohols (PVOH) are fossil‐based synthetic polymers that are water soluble and biodegradable. In 2022, the global market size for PVOH was US$1.0B. Kuraray, one of the producers of PVOH, has published the results of a life cycle assessment (LCA) for the production and biodegradability of PVOH providing details of their carbon and water footprint.^[^ ^23^ ^]^ In the LCA summary, Kuraray states that “be informed that the biodegradability of PVOH is associated with a renewed CO_2_ emission of 2 kg CO_2_ per kg PVOH, regardless of which PVOH it is.” In other words, non‐biogenic (i.e. fossil) CO_2_ will be released into the environment at the end‐of‐life of PVOH via biodegradation contributing to climate change. Therefore, the recirculation indicator should only be applied to biobased chemical products and not to fossil‐based products, i.e. only to products that are truly *re*circulated.

The ISO59020 also defines additional circularity indicators and one of them – nutrient extracted from discharged water – is relevant to the chemical industry (Table 2). The standard states that “any surplus nutrients and related materials that have been introduced to the water flow within the system in focus should be removed from the water during its recirculation or at the point of discharge. The extracted nutrients can be valuable resources and can be evaluated for further use. Nutrients include, for example, nitrate, phosphorous as phosphate, metals (e.g., Ca, Fe, Na, K), carbohydrates (e.g., lipids, fats, sugars), proteins, and vitamins.” Therefore, this circularity indicator calculates the percentage of materials recovered from wastewater at end‐of‐life that are redirected to production.

Next to the new circularity indicator that assesses the percentage of safe chemicals released over all chemicals released, a circularity indicator is needed that addresses the challenge of climate change. The chemical sector has the potential to reduce the CO_2_ concentration in the atmosphere by permanently sequestering CO_2_ into their products (Table 2). Importantly, the sequestering needs to be calculated over the total life cycle of the product (cradle‐to‐grave) and needs to have a net benefit.

## 7 Bioeconomy

The bioeconomy, i.e., an economy that uses biotechnology as well as biomass, is hailed as a solution to replace fossil‐based materials and supports a sustainable future. However, the industrial use of biomass affects biodiversity. As pointed out by Richardson et al.^[^ ^4^ ^]^ there are two planetary boundaries that need to be especially considered when discussing the impact of the bioeconomy on planetary health, i.e. the Functional Biosphere Integrity and the Land Use Change boundary. Importantly, both planetary boundaries are currently transgressed. In their work, the biosphere functional integrity is measured as energy available to ecosystems, i.e., net primary production (NPP). Consequently, Richardson et al. suggest using a new control variable, i.e., Human Appropriation of Net Primary Production (HANPP), to evaluate human biomass use. They set the upper limit of HANPP at 20%, however, state that HANPP is currently at 30% (**Table** **3**). Richard et al. also highlight that the increasing usage of biomass to replace coal, oil, and gas increases the pressure on the already transgressed Land Use Change boundary.^[^ ^4^ ^]^

**Table 3:** Planetary boundaries that are strongly affected by the bioeconomy.^[^ ^4^ ^]^

Earth system process	Control variable(s)	Planetary boundary	Preindustrial Holocene base value	Upper end of zone of increasing risk	Current value of control variable
Biosphere functional integrity: measured as energy available to ecosystems (NPP) (%HANPP)	HANPP (in billion tonnes of C year^−1^) <10% of preindustrial Holocene NPP, i.e., >90% remaining for supporting biosphere function	1.9% (2σ variability of preindustrial Holocene century‐mean NPP)	0 Tg of P year^−1^	20% HANPP	30% HANPP
Land System Change	*Global*: area of forested land as the percentage of original forest cover; *biome*: area of forested land as the percentage of potential forest (% area remaining)	*Global: 75%* values are a weighted average of the three individual biome boundaries; *biomes*: tropical, 85%; temperate, 50%; boreal: 85%	100%	*Global*: 54%; *biomes*: tropical, 60%; temperate, 30%; boreal: 60%	*Global*: 60%; *tropical*: Americas, 83.9%; Africa, 54.3%; Asia, 37.5%; *temperate*: Americas, 51.2%; Europe, 34.2%; Asia, 37.9%; *boreal*: Americas, 56.6%; Eurasia: 70.3%

## 8 Do We Need a Scope 1, 2, 3 Waste Framework?

Transparency of the environmental impact of a product or service is an important concept within the sustainability framework and is achieved through the calculation and publication of so‐called footprints. Best known is the carbon footprint expressed in kg CO_2_ eq. In addition, the Greenhouse Gas Protocol^[^ ^24^ ^]^ distinguishes between Scope 1 (direct emissions), and Scope 2 and 3 (indirect) carbon emissions.

One of the reasons for the currently very low circular material use rate value is the amount of waste generated along the lifecycle of a product as depicted in Figure 1. Enhancing the transparency for waste generation along the lifecycle and value chain of a product might lead to the reduction of waste generation and better use of waste. The problem of waste generation is already described by the material footprint or ‘ecological rucksack’ and resources for calculating the material footprint are available.^[^ ^25^ ^]^ Therefore, turning the material footprint or ecological rucksack into a mandatory reporting tool for the industry sector could support identifying hotspots and provide consumers with product transparency. A ‘waste framework’ could be established similar to the carbon emission reporting framework with Scope 1 (waste from resource extraction), Scope 2 (waste during production), and Scope 3 (waste during use and at end‐of‐life).

## 9 Conclusion

Overall, there is an urgent need for the chemical industry to become more circular. Transparency is an important tool to achieve a circular chemical industry. A bespoken set of circular economy indicators for the chemical industry sector should be developed as presented in this work and subsequently applied addressing all nine planetary boundaries in conjunction with targets that will support the return of the seven transgressed planetary boundaries to their safe planetary operating space. If circular economy principles are adapted widely and are carefully implemented, they will contribute significantly to preserving our natural environment for the current generation and generations to come. Concerted efforts are needed by all stakeholders along the value chain of the chemical industry as well as politics. After all, we are living in an area of global challenges.

## Conflict of Interest

The authors declare no conflict of interest.
